# Assessing the Effectiveness of a Multicomponent Intervention on Hand Hygiene and Well-Being in Primary Health Care Centers and Schools Lacking Functional Water Supply in Protracted Conflict Settings: Protocol for a Cluster Randomized Controlled Trial

**DOI:** 10.2196/52959

**Published:** 2024-04-03

**Authors:** Anaïs Galli, Yaman Ma'ani Abuzahra, Carola Bänziger, Aboubacar Ballo, Max N D Friedrich, Karin Gross, Miriam Harter, Jan Hattendorf, Maryna Peter, Andrea Tamas, Branwen N Owen, Mirko S Winkler

**Affiliations:** 1 Department of Epidemiology and Public Health Swiss Tropical and Public Health Institute Allschwil Switzerland; 2 Faculty of Natural Sciences University of Basel Basel Switzerland; 3 Faculty of Medicine and Health Sciences An-Najah National University Nablus Occupied Palestinian Territory; 4 Institute for Ecopreneurship University of Applied Sciences and Arts, Northwestern Switzerland (FHNW) Muttenz Switzerland; 5 WASH Regional Department Africa Terre des hommes Bamako Mali; 6 Ranas Zurich Switzerland

**Keywords:** water, sanitation, and hygiene, WASH, hand hygiene, impact evaluation, conflict settings, behavior change, handwashing, students, handwashing stations, primary schools, primary health care facilities, humanitarian crisis, mobile phone

## Abstract

**Background:**

Hand hygiene is crucial in health care centers and schools to avoid disease transmission. Currently, little is known about hand hygiene in such facilities in protracted conflict settings.

**Objective:**

This protocol aims to assess the effectiveness of a multicomponent hand hygiene intervention on handwashing behavior, underlying behavioral factors, and the well-being of health care workers and students. Moreover, we report our methodology and statistical analysis plan transparently.

**Methods:**

This is a cluster randomized controlled trial with 2 parallel arms taking place in 4 countries for 1 year. In Burkina Faso and Mali, we worked in 24 primary health care centers per country, whereas in Nigeria and Palestine, we focused on 26 primary schools per country. Facilities were eligible if they were not connected to a functioning water source but were deemed accessible to the implementation partners. Moreover, health care centers were eligible if they had a maternity ward and ≥5 employees, and schools if they had ≤7000 students studying in grades 5 to 7. We used covariate-constrained randomization to assign intervention facilities that received a hardware, management and monitoring support, and behavior change. Control facilities will receive the same or improved intervention after endline data collection. To evaluate the intervention, at baseline and endline, we used a self-reported survey, structured handwashing observations, and hand-rinse samples. At follow-up, hand-rinse samples were dropped. Starting from the intervention implementation, we collected longitudinal data on hygiene-related health conditions and absenteeism. We also collected qualitative data with focus group discussions and interviews. Data were analyzed descriptively and with random effect regression models with the random effect at a cluster level. The primary outcome for health centers is the handwashing rate, defined as the number of times health care workers performed good handwashing practice with soap or alcohol-based handrub at one of the World Health Organization 5 moments for hand hygiene, divided by the number of moments for hand hygiene that presented themselves during the patient interaction within an hour of observation. For schools, the primary outcome is the number of students who washed their hands before eating.

**Results:**

The baseline data collection across all countries lasted from February to June 2023. We collected data from 135 and 174 health care workers in Burkina Faso and Mali, respectively. In Nigeria, we collected data from 1300 students and in Palestine from 1127 students. The endline data collection began in February 2024.

**Conclusions:**

This is one of the first studies investigating hand hygiene in primary health care centers and schools in protracted conflict settings. With our strong study design, we expect to support local policy makers and humanitarian organizations in developing sustainable agendas for hygiene promotion.

**Trial Registration:**

ClinicalTrials.gov NCT05946980 (Burkina Faso and Mali); https://www.clinicaltrials.gov/study/NCT05946980 and NCT05964478 (Nigeria and Palestine); https://www.clinicaltrials.gov/study/NCT05964478

**International Registered Report Identifier (IRRID):**

DERR1-10.2196/52959

## Introduction

### Background

Globally, approximately 3% of deaths and 5% of disability-adjusted life years are attributable to the effects of a lack of safe water, sanitation, and hygiene (WASH) [1]. More than half of WASH-attributable deaths occur in sub-Saharan Africa [1].

Hand hygiene is crucial to sustaining individual and community health. Pathogens can easily be transmitted from contaminated hands to other people’s hands, eyes, and mouths [2]. Inadequate hand hygiene can therefore increase the risk for transmission of diarrheal, respiratory, and skin diseases [3-5].

In 2022, a quarter of people worldwide [6] and three-quarters of people living in sub-Saharan Africa [7] lacked access to basic hygiene, defined as having a functioning handwashing station on the premises with water and soap. Hand hygiene is of particular importance in health care centers and schools (which we collectively refer to here as facilities) because populations considered vulnerable frequent them. In these facilities, pathogens can spread easily over health care workers’ or students’ hands [8,9]. In health care centers, basic hygiene means additionally that the handwashing station needs to be at the point of care and within 5 m of toilets, while it can be equipped with hand sanitizer instead of soap [10]. Access to basic hygiene in both facility types is low. Only three-fifths of schools worldwide [11] and two-fifths of health care centers at the point of care in low- and middle-income countries [10] are estimated to have access to basic hygiene services. Handwashing stations are half as common in primary health care centers compared to secondary and tertiary health care centers [12].

In fragile and conflict settings, sanitation infrastructure is often overburdened, water quality is poor, and water quantity is insufficient [13,14]. Water is thus primarily used for drinking and cooking, and hygiene and sanitation needs become secondary [15,16]. Basic hygiene access in health care centers and schools is therefore of even greater importance. There are little data on handwashing practices in these facilities, although the United Nations Children’s Fund estimates that >50% of children without basic hygiene at school live in fragile or conflict contexts [17]. Such facilities are often excluded from hand hygiene investigations during crises, despite the increased risk of infections [18,19]. Furthermore, collecting baseline data and following monitoring and evaluation activities of hand hygiene usually have a low priority in these facilities [14,18,19].

### Objectives

Our study is embedded in the hands4health (h4h) project as an effectiveness evaluation component. The project is funded by the Swiss Agency for Development and Cooperation and led by 10 partners from academia, nongovernmental organizations, and the private sector [20] in close collaboration with the ministries of health (MoHs), ministries of education (MoEs), and other key stakeholders of the project countries, Burkina Faso, Mali, Nigeria, and Palestine (refer to Multimedia Appendix 1 for the project consortium overview). The h4h project has the overarching objective of increasing hygiene in primary health care centers and schools without any functional water supply in the context of protracted conflict settings. The h4h consortium developed a systematic approach with a multicomponent hand hygiene intervention (MCHHI; refer to the Intervention and Control subsection) to improve the health of patients, health care providers, students, and teachers by improving their hand hygiene and water infrastructure. Our study aims to evaluate the effectiveness of the h4h MCHHI on the hand hygiene of health care workers and students by achieving objectives 1 to 4 (Textbox 1). All our objectives will be assessed using a cluster randomized controlled trial (cRCT) design because the MCHHI is implemented at cluster level (ie, health care center or school). By publishing this protocol, we aim to transparently report our study design, methods, and statistical analysis plan. Consequently, we intend to avoid duplicate studies, coordinate research efforts, and demonstrate our accountability to report the results in a timely manner.

**The hands4health (h4h) study aim with the more specific objectives 1 to 4.**

**Evaluate the effectiveness of the h4h multicomponent hand hygiene intervention (MCHHI) on the hand hygiene of health care workers and students**

- Objective 1: assess the effectiveness of the h4h MCHHI on the hygiene-related risks, attitudes, norms, abilities, and self-regulation (RANAS) behavioral factors; handwashing behavior; and well-being of health care workers and students.
- Objective 2: assess the hygiene-related RANAS behavioral factors, handwashing practices, and well-being of health care workers and students at baseline.
- Objective 3: assess the perceived effectiveness of the h4h MCHHI on the health and well-being of health care workers and students in the intervention facilities.
- Objective 4 (exploratory): assess the effectiveness of the h4h MCHHI on the predefined health conditions and absenteeism of health care workers and students (objective 4 is exploratory because we did not take it into account during the sample size calculation; therefore, the sample size might be too small to detect a difference in the incidence of hygiene-related health conditions between the 2 study arms; however, as patient information remains anonymous, and the data collection does not add a big burden to the implementation partners, we decided to include this objective).

## Methods

### Study Design

**Figure 1:**
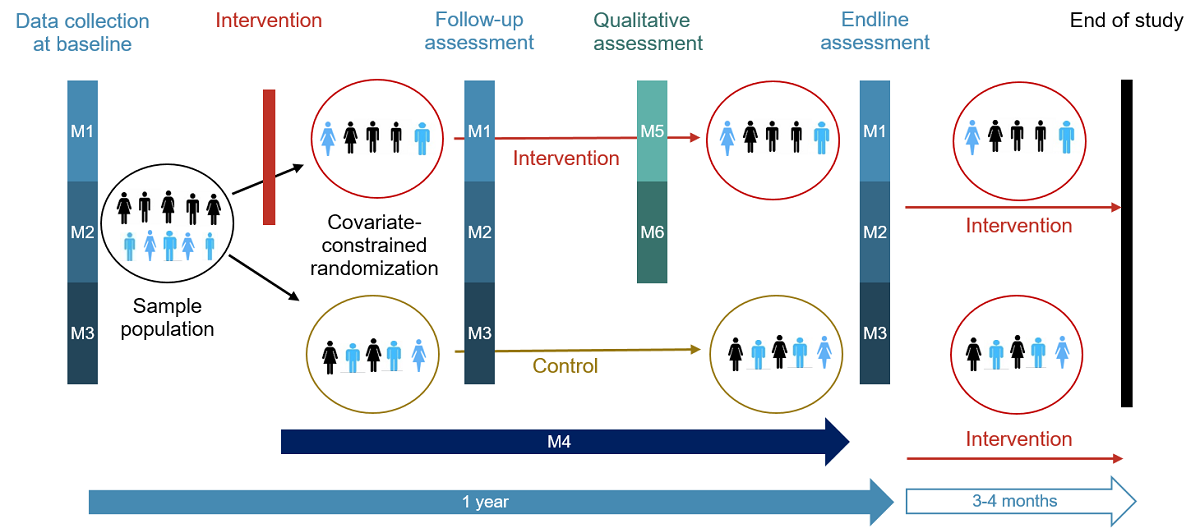
Overview of the hands4health study design, including the data collection methods M1 to M6. M1: module 1 (risks, attitudes, norms, abilities, and self-regulation and well-being survey); M2: module 2 (structured observation); M3: module 3 (hand-rinse samples); M4: module 4 (diary approach); M5: module 5 (focus group discussions); and M6: module 6 (key informant interviews).

We are conducting a parallel cRCT in 4 countries (Burkina Faso, Mali, Nigeria, and Palestine) with 2 study arms per country (Figure 1). For the cRCT, we collected baseline data in 24 primary health care centers per country (March to May 2023 in Burkina Faso and Mali) and 26 schools per country (February to June 2023 in Nigeria and Palestine). The data collection included hand hygiene observations; a survey about hand hygiene-related risks, attitudes, norms, abilities, and self-regulation (RANAS) behavioral factors as well as practices and well-being; and microbiological analyses of hand-rinse samples. The project partners implemented the h4h MCHHI in 12 randomly assigned primary health care centers and 13 randomly assigned schools per country. One to 2 months after the implementation of the intervention, we conducted follow-up data collection in both study arms where we repeated the observations and the survey, plus additional qualitative data in the respective intervention arms (November to December 2023 in all 4 countries). One year after baseline, we collect endline data in both study arms, including the same types of quantitative data as in the baseline data collection period (February to April 2024 in Burkina Faso and Mali and May-June 2024 in Nigeria and Palestine).

We use a mixed methods design [21,22]. By collecting qualitative data before the start of the cRCT and during the cRCT, we assess WASH-related needs and perceptions about the effectiveness of our intervention on the health and well-being of our study population. By collecting quantitative data about WASH-related health determinants such as the prevalence of handwashing and RANAS behavioral factors, we will assess the effectiveness of the h4h MCHHI on our study population’s hand hygiene and well-being.

Our study design is based on previous h4h project activities. First, the project coordination partner commissioned 2 reviews on current tools used for WASH infrastructure in health care centers and schools [23]. Second, the main local implementation partners used a Facility Evaluation Tool for Water, Sanitation, and Hygiene in Institutions (FACET) to assess the WASH infrastructure of the facilities in the study regions [24]. Third, the project coordination partners led a theory of change approach [25]. The theory of change aims to understand the complexity of the WASH system in the study countries to identify key stakeholders, problems, and starting points for potential solutions. The partners conducted various workshops and interviews with stakeholders and project staff to support this process. Fourth and last, we collected qualitative data with focus group discussions (FGDs) to investigate the local perceptions and needs of hygiene in the pilot facilities. The project consortium then used all this information to develop the h4h MCHHI for each country (refer to Multimedia Appendix 2 [26-37]).

### Setting

This study focuses on primary health care centers in rural areas of Burkina Faso and Mali and primary schools in Nigeria and Palestine from 2021 to 2024 (Table 1). All 4 countries involved in this study are subject to protracted conflicts. In the West African countries, jihadists and other radical groups have been terrorizing the population, leading to a rise in internally displaced persons, while Palestine has been under Israeli military occupation for 56 years [38-41]. All targeted facilities in our study regions suffer severe water shortages and a lack of WASH infrastructure and maintenance activities. Moreover, most facilities expect being overcrowded in the near future due to a rise in internally displaced persons. In Maiduguri, Borno State, Nigeria, the schools are already overcrowded, with an average of 101 students per elementary class [42].

**Table 1:** Description of the country settings of the hands4health project.

	Burkina Faso	Mali	Nigeria	Palestine
Cluster type	Primary health care centers	Primary health care centers	Primary schools	Primary schools
Clusters (n=100), n (%)	24 (24)	24 (24)	26 (26)	26 (26)
Geographic region	West Africa	West Africa	West Africa	Middle East
Country region	Boucle du Mouhon	Ségou and San	Borno State	West Bank
Districts	Dédougou and Boromo	Macina, Markala, San, and Tominian	Maiduguri	Hebron
Reasons for instability	Occupation by jihadist and other radical groups; 2 coups d’états and a political crisis	Occupation by jihadist and other radical groups; 2 coups d’états	Boko Haram insurgency	Israeli military occupation
Internally displaced people per country, n	2.06 million^a^	375,500^b^	2.2 million^c^	N/A^d^

^a^Burkina Faso: Aperçu des personnes déplacées internes (Overview of internally displaced persons; United Nations Office for the Coordination of Humanitarian Affairs [OCHA]; March 31, 2023) [43].

^b^Mali: Tableau de bord humanitaire (Humanitarian dashboard; OCHA; April 30, 2023) [44].

^c^Nigeria: Situation Report (OCHA; July 18, 2023) [45].

^d^N/A: not applicable.

### Intervention and Control

**Figure 2:**
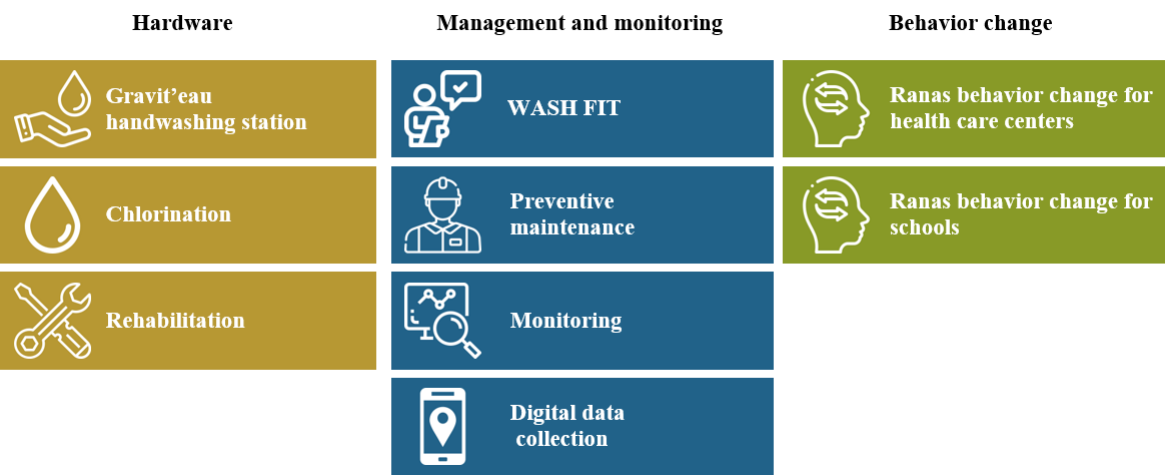
The hands4health multicomponent hand hygiene intervention components implemented in at least one of the study countries grouped according to hardware (yellow), management and monitoring (blue), and behavior change (green). The icons were sourced from Freepik. RANAS: risks, attitudes, norms, abilities, and self-regulation; WASH FIT: Water and Sanitation for Health Facility Improvement Tool.

The MCHHI varies by study country because it was adapted to each country’s needs, local acceptability of intervention components, and tools available for health care centers or schools (refer to Multimedia Appendix 2 for a detailed description). The intervention components, covering hardware, management and monitoring, and behavior change are depicted in Figure 2. In terms of hardware, a handwashing station that recycles water, called Gravit’eau, was locally built and adapted in terms of height and size of basin to the type of institution (ie, health care center or school) in the African countries. Per health care center, 2 individual stations were installed and positioned according to the health care provider’s wishes. In Nigeria, 2 stations were placed near 4 to 6 classrooms and toilet areas serving our selected sample of 50 students in each school. In Palestine, recycled water was not culturally accepted. Therefore, instead of receiving a station, we supported the schools with infrastructure rehabilitation work. Facilities in the control arm will receive the intervention once the cRCT is completed. Any viable potential improvements identified during the trial will be implemented.

### Recruitment and Eligibility

#### Primary Health Care Centers

We selected the primary health care centers with the support of the local implementation partners. They conducted a FACET survey in 179 centers in Burkina Faso and 60 centers in Mali. From these centers, we selected a subset based on the following inclusion criteria: at the time of the FACET survey, the facility (1) did not have any water source directly connected to the building, (2) had a maternity ward, (3) had at least 5 employees, and (4) was impacted by insecurity but was still deemed accessible to the implementation partners. Of the centers meeting these criteria, the implementation partners chose 48 (n=24, in each country) that were most likely to remain accessible for at least 1 year.

As study participants in the health care centers at baseline, we invited all health care workers who met the inclusion criteria and were present at the time of data collection for the quantitative data collection. The inclusion criteria for the quantitative data collection were as follows: health care workers (1) aged at least 18 years and (2) in direct physical contact with patients. The exclusion criteria were as follows: health care workers (1) whose primary occupation does not involve the health care center of the h4h project, (2) who have a skin condition that precludes the use of soap or alcohol-based handrub, and (3) who refuse to participate. All recruited participants received a unique ID. The same eligibility criteria will apply for the follow-up (1-2 months after the intervention) and endline (12 months from the baseline) data collection.

As part of the quantitative data collection in health care centers, we conducted structured observations during patient consultations. The patients needed to fulfill the following inclusion criteria: (1) aged at least 18 years or being accompanied by a legal guardian aged ≥18 years; and (2) visiting the facility for a physical examination, injections or vaccinations, or a blood test. We did not observe sensitive procedures such as giving birth.

Participants for the qualitative data collection at follow-up will be recruited from those who participated in the quantitative baseline data collection. In addition, we will seek to recruit others working in the intervention centers, such as hygiene technicians or people working in administrative positions, as well as stakeholders within the community, state, region, or country of the intervention whose position is related to WASH in health care centers.

#### Primary Schools

The MoE in Nigeria and Palestine identified 51 and 50 schools, respectively, as being greatly in need of WASH infrastructure. The implementation partners conducted a FACET survey in each of these schools before the start of this study. From these 101 schools, we selected 52 (51.5%; n=26, in each country) based on the following inclusion criteria: at the time of the FACET survey, the school (1) was deemed accessible to the implementation partners, (2) lacked a functional water source, and (3) had ≤7000 students studying in grades 5 to 7.

For the baseline data collection, we selected 50 eligible students aged 10 to 12 years from 1 or 2 classes from each school using random sampling. If a school had a single class consisting of ≥50 students within our target age group, we selected our sample from this class. However, if no class in our target age group had ≥50 students, we randomly selected 2 classes from within the target age group and selected our sample from these classes. For the different data collection modules, we randomly selected subsets from the 50 previously selected students (Figure 1). The inclusion criteria for both quantitative and qualitative data collection were as follows: (1) aged 10 to 12 years and (2) registered at the school for the duration of the study period (1 year). The exclusion criteria included (1) not providing signed consent (for the guardians) and assent form (for the students), (2) having a medical condition that prevents them from washing their hands, (3) having unexplained intermittent attendance in school (school teachers were consulted, and the school’s absenteeism records were checked), and (4) not being in the same school for the course of the study (1 year). The same 50 students will be revisited for the follow-up (1-2 months after the intervention) and endline (12 months from the baseline) data collection.

In addition to the data collected from students, we will collect qualitative data from teachers and key stakeholders at follow-up. The eligibility criteria for teachers are as follows: (1) aged ≥18 years, (2) permanent employee in one of the intervention schools, and (3) teaching one of the classes included in the intervention. The exclusion criterion is refusal to participate. Key stakeholders will be identified through purposeful sampling, primarily through consultation with the local implementation partners. Key stakeholders will be eligible for inclusion in the study based on the following criteria: (1) they occupy a role within the community, state, or region of the study that is associated with WASH in schools (eg, teachers and school principals or people working in any of the organizations that are part of the WASH cluster, such as the MoE or MoH); and (2) they are aged ≥18 years. The exclusion criterion is refusal to participate.

### Ethical Considerations

All our study protocols were approved by the Ethikkommission Nordwest- und Zentralschweiz (Ethics Committee for Northwestern and Central Switzerland; AO_2023-00004 and AO_2023-00047) and the ethics boards in each project country: (1) the Comité d’Ethique pour la Recherche en Santé (Ethics Committee for Health Research) in Burkina Faso (2023-02-020), (2) the National Institute of Public Health in Mali (05/2023/CE-INSP), (3) the National Health Research Ethics Committee in Nigeria (21/2023), and (4) the institutional review board of An-Najah National University in Palestine (H Sp. Feb. 2023/18). Study participants in the health care centers and legal guardians of schoolchildren gave written informed consent to participate in this study, whereas schoolchildren gave oral assent. For structured observations, the local implementation partners obtained consent in both facility types before the baseline observations. At the health care center level, consent was obtained from the director of the center for the duration of the study period. In addition, oral consent was obtained from the patients who visited the centers at the time of the observations. Similarly, at the school level, consent was secured from the school administration and from the parents of the participating students. All participants received a unique ID that was coded and will be completely anonymized by the end of this study. Our study protocols for health care centers and schools are registered in ClinicalTrials.gov (NCT05946980 and NCT05964478). We report our study in line with the CONSORT (Consolidated Standards of Reporting Trials) statement: extension to cluster randomized trials [46].

### Randomization

We used covariate-constrained randomization to allocate facilities into the control and intervention arms. This methodology, also referred to as restricted randomization, was first suggested by Moulton [47] to minimize the risk of baseline imbalances. These issues are usually more prominent in cRCTs due to the relatively low number of randomized units.

A statistician who was not involved in any h4h field activities performed the covariate-constrained randomization after baseline data collection following these steps: (1) definition of a set of maximum imbalance criteria for several important baseline characteristics, (2) generation of a list of all potential allocation sequences that satisfy these criteria, (3) verification of the independence of units, and (4) random selection of one of the valid sequences as the final allocation.

For health care facilities in Mali and Burkina Faso, we defined the following four constraints: (1) proportions of health care facilities with shortages in water sources should be perfectly balanced, (2) a difference in the proportion of secure facilities of <10 percentage points, (3) a difference in the mean proportions of the population living within 5 km of <10 percentage points, and (4) a difference in the mean proportions of accurate handwashing in each facility of <3 percentage points. In Mali and Burkina Faso, 248,574 and 767,792 potential allocation sequences, respectively, satisfied all 4 criteria.

For schools in Palestine, we first stratified the clusters before applying the restricted randomization. This stratification was based on two key parameters: (1) the directorate in which the school is located; and (2) the school’s geographic location, categorized as city, town, or village. After stratification, the allowed allocation sequences had to satisfy the following criteria: (1) balanced administrative areas for schools across both arms, (2) balanced sex, (3) a difference of ≤33% points in the proportions of schools connected to a water network across the arms, (4) a difference of ≤33% percentage points in the proportions of schools with consistent access to water, (5) a difference of <50 in the mean numbers of students across the arms, (6) a difference of <0.2 in the mean grades across the arms, and (7) a difference of <50 in the mean ratios of the number of handwashing stations to the total number of students across the arms. In total, 394 potential allocation sequences satisfied all criteria.

For Nigeria, we defined the following criteria: (1) similar proportions of schools with no currently available water source, (2) similar proportions of schools with <2 days of water availability, (3) similar proportions of schools located in a village as opposed to a city or town, and (4) similar proportions of primary schools in comparison to mixed primary and secondary schools. Other variables allowed a certain degree of imbalance but still required consideration to ensure appropriate balance. These included (1) a difference of ≤200 students in the average student numbers per school between the study arms, (2) a difference of ≤25 percentage points in the proportions of schools with water network connections across the arms, and (3) a difference of ≤25 percentage points in the proportions of schools with water availability for 3 to 5 days a week across the arms. Of all potential allocation sequences, 57,054 satisfied all criteria.

### Blinding

To reduce selective counting of colony-forming units (CFUs) of bacteria in the hand-rinse samples collected at baseline, the laboratory workers assessing the number of CFUs were blinded. The data collectors delivered the coded samples to the laboratory workers. From the identification code of the samples, the laboratory workers could not derive whether the sample was collected in an intervention facility or a control facility. The laboratory workers will be blinded again at endline data collection.

### Quantitative Methods

We collected quantitative data with four different modules: (1) module 1 (RANAS and well-being survey), (2) module 2 (structured handwashing observations), (3) module 3 (microbiological analysis of hand-rinse samples), and (4) module 4 (diary approach for predefined health conditions).

#### Module 1: RANAS and Well-Being Survey

We started the survey by asking participants about sociodemographic information, followed by knowledge questions about hand hygiene and self-reported hand hygiene practices (for detailed descriptions of the variables, refer to Multimedia Appendix 3). The survey proceeded with questions to measure the RANAS behavioral factors, such as perceptions of costs and benefits of consistent hand hygiene, social norms, and ability beliefs, on 5-point Likert scales to measure frequencies, magnitudes, and degrees [27]. Questions about the hygiene infrastructure in the facilities followed this section. In health care centers, there were additional survey items on the quality of care, and in schools, on student well-being. Well-being was assessed with the KINDL tool (Kinder Lebensqualität Fragebogen, Children’s Quality of Life Questionnaire), which was developed specifically to measure health-related quality of life in children and adolescents by assessing different domains of well-being [48].

We conducted the survey with all participating primary health care workers and a randomly chosen subset of 25 students per school at baseline and will repeat at follow-up (1-2 months after the intervention) and endline (12 months from the baseline). The survey will be interviewer administered using Open Data Kit Central software (version 2022.3.1) on Android tablets or smartphones.

#### Module 2: Structured Handwashing Observations

Structured handwashing observations are considered the gold standard method to assess handwashing behavior [12,49]. We conducted the observations with primary health care workers and schoolchildren at baseline and will repeat these observations at follow-up and endline. Due to structural differences in health care centers and schools, we chose 2 different observation approaches for the 2 facility types.

In primary health care centers, staff members from the implementation partner or the regional MoH were trained by the first author (AG) as observers for the baseline data collection. Observers usually worked in the health care centers in a different role, and they visited the facility under the pretext of performing their usual role and did not declare that they were observing handwashing. Hence, the handwashing observations were conducted covertly. The observers were equipped with an observation tool programmed in Open Data Kit Central (version 2022.3.1) on their smartphone. During their visit, they spent 1 hour in each unit to record all hand hygiene actions taken by the health care providers, as well as their handwashing techniques [50]. The same observation process will be repeated at follow-up and endline.

In schools, student handwashing was observed by implementation partners who were trained by the second author (YA). To aid the identification of participating students, all children were given badges, with 1 color for those participating and another for those not participating. The students were told that the color assignment was random. The implementation partners observed each student’s handwashing practices across a variety of occasions, including the 2 critical moments: before eating and after using the toilet (among others, eg, after eating and after playing). To observe handwashing before eating, we involved the students in an experiment. We engaged the children in a 30-minute painting activity, after which the children were rewarded with a popcorn snack and granted a 15-minute break to enjoy it together. This created an opportunity to observe whether the children washed their hands before eating the snack. Observers did not reveal their intention of observing handwashing throughout the time of the data collection. In Nigeria, we additionally observed the children for 3 hours. The observation started at 9 AM (60 minutes before the breakfast break) and ended at noon (90 minutes after the break). Four observers were stationed near all available water points and toilets. Using a paper-based observation tool, the observers recorded all participating students who washed their hands and whether they did so before eating, after using the toilet, or at another time point. In addition, any participating student observed using the toilet who did not wash their hands was also recorded.

Observers then cross-checked their results to eliminate instances of duplicate observations for the same students. They merged observations made at different critical times or water points for the same student and confirmed whether those who used the toilet also washed their hands, particularly in scenarios where toilets were situated at a considerable distance from the water points. After this cross-checking process, the data were transferred to Open Data Kit Central (version 2023.3.1) for analysis and storage. These observations yielded a dichotomous measure: whether handwashing occurred at each critical event. However, options such as “not visible” or “soap or water was not available” were included to account for potential complications. If feasible, we observed the handwashing steps of students who washed their hands. Observations will be repeated in schools at follow-up and endline.

#### Module 3: Microbiological Analysis of Hand-Rinse Samples

We collected hand-rinse samples of all participating health care workers and a randomly selected subset of 12 students at baseline with a modified glove juice method as described by Pickering et al [51]. We asked the participants to insert their hand into a Whirl-Pak bag (Nasco Sampling LLC; sizes 2041 mL and 7120 mL for adults and children, respectively), filled with 350 mL of bottled drinking water without chlorine. The participants were asked to shake their hand in the water and rub their thumb and fingers together for 15 seconds, and then the sample collector massaged the participant’s hand through the bag for another 15 seconds [51]. Afterward, we repeated the procedure with the other hand.

We kept the Whirl-Pak bags containing the samples on ice in an isolation box and processed them within 8 hours of sampling [51]. We used membrane filtration to detect CFUs of *Escherichia coli* and total coliforms. In a field laboratory, we passed 100 mL of the bag’s content through the filter paper, which we then placed on Nissui Compact Dry EC plates (Shimadzu Diagnostics Europe) to incubate them at 35 °C −0.5 °C to +0.5 °C for a duration of 24 hours [51]. For quality control, we carried out a duplicate filtration of every 10th sample and a negative control, only containing the bottled water, each day.

We calculated the lower detection limit of CFUs by dividing 1 CFU per plate by the filtrate volume and then multiplying it by the total Whirl-Pak volume of 350 mL. We calculated the upper detection limit by dividing 301 CFUs per plate by the filtrate volume and then multiplying it by the Whirl-Pak volume. We normalized and log_10_-transformed the CFUs per hand for the statistical analysis [51]. We will repeat the same hand-rinse sampling procedure again at follow-up and endline.

#### Module 4: Diary Approach for Predefined Health Conditions

Using a diary approach, we will ask health care center directors to collect longitudinal data on the hygiene-related health conditions of patients. These conditions include maternal and neonatal mortality, stillbirths, postpartum endometritis, neonatal sepsis, umbilical cord infections, and infections of wounds after treatment. For each relevant health condition among patients, the director will be asked to report the date of diagnosis, and if known, the duration of the condition, the etiology, and ultimate outcome (eg, recovery or death). Detailed descriptions of the health conditions are presented in Multimedia Appendix 4 [52].

Similarly, hygiene-related absences of all participating health care workers and the 50 students will be reported during the period after the intervention until the endline data collection in both study arms. For absenteeism in health care centers or schools, the person responsible will record the event, the number of days absent, and the reason for the absence. We define absenteeism as hygiene related if the facility worker or student experienced diarrheal diseases (including cholera), respiratory tract infections (including tuberculosis, COVID-19, and influenza), bacterial infections of the skin and eyes (including trachoma), and newly diagnosed HIV and hepatitis B or C. The diary will consist of 1 table per month containing all different health conditions and subcategories. No personal information will be recorded in this module.

### Outcomes

#### Primary Outcome

Due to the difference in settings, health care centers and schools have different primary outcomes. In health care centers, the primary outcome is the handwashing rate, defined as the number of times each health care worker performs good handwashing practice with soap or alcohol-based handrub at one of the World Health Organization (WHO) 5 moments for hand hygiene [53], divided by the number of moments for hand hygiene that presented themselves during the patient interaction. The handwashing rate was assessed by structured handwashing observations over 1 hour per unit in a health care center. The 5 moments for hand hygiene are defined by the WHO as follows:

1. Before touching a patient
2. Before clean or aseptic procedures
3. After risk or exposure to body fluids
4. After touching a patient
5. After touching a patient’s surroundings

In schools, the primary outcome is the number of participating students who wash their hands before eating. This number was collected using structured handwashing observations after students were given a snack after their participation in a painting activity. We selected this outcome because it allows for an objective assessment across all participating students, given that all were presented with this handwashing opportunity during the course of the experiment.

#### Secondary Outcomes

The most important secondary outcomes in both settings are as follows:

1. Self-reported handwashing practice on a Likert scale ranging from *almost never* to *almost always* (in health care centers: for each of the 5 moments for hand hygiene; in schools: before eating and after using the toilet)
2. The log_10_-transformed number of total coliforms and *E. coli* CFUs per hand before handwashing
3. RANAS behavioral factors measured on a 5-point Likert scale
4. Hygiene-related absenteeism and health conditions, which are summarized in Multimedia Appendix 4; the sum of each outcome variable will be used separately per facility (cluster) as a measure for statistical analysis, and health conditions will be reassessed with local experts in the respective countries to ensure feasibility before the start of the intervention

Secondary outcomes only applying to the school setting are as follows:

1. Good handwashing practice defined as the number of students who wash their hands after using the toilet (assessed by structured handwashing observation)
2. Self-reported well-being of students assessed using the KINDL tool [48], with responses given on a Likert scale ranging from *almost never* to *almost always*

### Statistical Analysis

#### Sample Size Calculation

We ran a series of simulations using R software (version 4.1.3; R Foundation for Statistical Computing) to determine the required sample size for health care centers. For the simulations, we assumed 6 staff members per health care center, a mean number of 5 (SD 5) times a person was supposed to wash their hands, and an intracluster correlation coefficient of 0.15; we needed to enroll 10 health care centers in each trial arm to detect a difference of 15 percentage points in the proportions of handwashing during the 5 critical moments for handwashing (30% control vs 45% intervention) with 81% power at a 2-tailed 5% significance level. To account for potential loss to follow-up, we enrolled 24 health care centers per country in Burkina Faso and Mali.

Within schools, we assessed the number of clusters (schools) and students per school with simulations using R. We aimed for a power of 80% at the 95% CI and anticipated an intraclass correlation coefficient of 0.2. In the simulation, we assumed a prevalence of handwashing before eating of 20% in control schools versus 45% in intervention schools after 1 year from the baseline. On the basis of this assumption, we needed 13 schools per trial arm with 50 students per school. However, the number of students assessed for different modules will differ throughout the study due to capacity limitations of assessing the 50 students included in each school.

#### Statistical Methods

We summarized baseline characteristics using descriptive statistics (Textbox 2). We will investigate the difference in the observed proportions of always handwashing at the 5 critical moments as defined by the WHO for primary health care centers and of the number of participants who wash their hands before eating for primary school students between the 2 study arms at follow-up using random effect logistic regression models. We will only include the intervention as a predictor in the primary analysis. For the primary analysis, we will use the available case population of health care workers and students, which will be analyzed according to the intent-to-treat principles.

**Statistical methods used for the analysis of the results of the different quantitative modules of the hands4health study.**

**Modules and statistical methods**

- Risks, attitudes, norms, abilities, and self-regulation and well-being survey (module 1)
  Descriptive statistics
  Random effect linear regression modeling
  Random effect logistic regression modeling
- Structured handwashing observations (module 2)
  Random effect logistic regression modeling
- Microbiological analysis of hand-rinse samples (module 3)
  Random effect negative binomial regression modeling
- Diary approach for predefined health conditions (module 4)
  Descriptive analysis with the sum of each outcome variable calculated separately per facility
  Random effect linear regression modeling

### Qualitative Methods and Analysis

Before the baseline data collection and at follow-up, we supplement our quantitative methods with two modules of qualitative methods: (1) module 5 (FGDs) and (2) module 6 (key informant interviews [KIIs]).

#### Module 5: FGDs

FGDs offer a practical way to gather insights more efficiently than other qualitative methods [54,55]. Before the onset of the study, the local implementation partners with experience in qualitative data collection collected data through FGDs involving health care workers, students, and teachers. These FGDs helped us to gain insight into the hygiene-related needs as well as the acceptability of our intervention among health care workers and students. We let the groups vote for their most urgent hygiene-related needs as well as the potential positive and negative impacts of our intervention. After counting and discussing the votes during the FGD, the project consortium adapted the country-specific interventions, and we identified what we needed to include in the quantitative data collection.

We collect data with FGDs again since January 2024 and will continue this data collection until April 2024 with the same trained implementation partners for health care workers and students to assess the perceived effectiveness and potential improvements of the h4h intervention. In addition, we will conduct FGDs with teachers from the schools in the intervention arm. We will train the team containing at least 1 moderator and 1 observer or notetaker before data collection. If the security situation allows, AG or YA will join the team. We will use a field research journal throughout the study to take structured notes and observations of the FGDs. We will audio record and transcribe the discussions and then translate into French or English for further analysis.

All health care workers in the intervention centers will be invited to participate in FGDs.

In health care centers, we will take into account the structures of hierarchy and gender when building mini groups of 3 to 4 participants to allow participants the greatest possible freedom of speech. We chose to have mini groups because the health care workers who participate are expected to have a high expertise in hand hygiene. Usually, the higher the expertise of participants, the smaller the group can be [54]. Depending on the hierarchical levels, 1 to 4 FGDs will take place per facility. We expect to conduct a maximum of 20 FGDs per country (fewer if we reach saturation beforehand) [55].

In schools, we will select students from each class in the intervention arm, in consultation with the school administration. We will conduct FGDs with 5 to 10 boys and girls separately until saturation is reached (or up to the maximum of 5 FGDs with each sex) and 5 FGDs with teachers [55]. KIIs may be conducted instead of FGDs if we do not have enough participants to form groups in both facility types.

#### Module 6: KIIs

Aiming to further explore issues arising from the FGDs and to understand perceptions of the intervention, we will conduct KIIs with other stakeholders who influence the project’s intervention areas, such as representatives of the MoH and MoE as well as local majors. We have identified some of these key informants through the theory of change approach, while others will be suggested by local partners. Here, we chose individual interviews rather than FGDs so that the stakeholder’s status does not influence other participants’ freedom of speech [55].

If necessary and appropriate, we may carry out interviews on the web, in French or English. Local project partners with experience in interviewing will conduct stakeholder interviews until saturation is reached [55]. We will apply the same steps of audio recording, transcription, and translation for the KIIs as for the FGDs.

We will analyze FGD and KII transcripts as well as field and observation notes using the framework method [56] with MAXQDA software (VERBI GmbH) with at least 1 local partner per country with qualitative expertise.

### Triangulation of Results

We triangulate our results throughout the study. Qualitative data from the pilot phase informed our quantitative data collection at baseline. Moreover, questions identified during the quantitative baseline and follow-up data collection will guide our qualitative FGDs and KIIs, which can then be used to better understand our results from the endline data collection By triangulating the results, we will be able to validate them, find discrepancies, and increase our understanding of why certain quantitative results emerged [21,22]. In addition, we might identify new research gaps and potential solutions to issues that we identified during the study period.

## Results

The baseline data collection of this study started in February 2023 and ended in June 2023. In Burkina Faso, we conducted 95 RANAS surveys, observed 82 participants, and collected 99 hand-rinse samples in 24 primary health care centers. In Mali, we conducted 105 RANAS surveys, observed 111 participants, and collected 100 hand-rinse samples in 24 primary health care centers. As of March 2024, the analysis of the baseline results in health care centers is completed and a manuscript will be submitted within the same month for publication. In Nigeria, we conducted 640 RANAS surveys, observed 1300 participants, and collected 369 hand-rinse samples in 26 primary schools. In Palestine, we conducted 646 RANAS surveys and observed 1127 participants in 26 primary schools. We did not collect hand-rinse samples in Palestine because the method was deemed culturally inappropriate by the MoE. As of March 2024, data analysis for schools is ongoing. Follow-up data collection took place from November to December 2023 in 24 health care centers in Burkina Faso and Mali, respectively and in 26 schools in Nigeria. Due to the crisis in Palestine, follow-up activities were suspended. As of March 2024, data analysis remains ongoing. Qualitative data collection started in Burkina Faso in January 2024 and is planned to start in Mali and Nigeria in March 2024. Endline data collection started in Mali in February 2024 and will be completed in all countries by June 2024. We will publish more results investigating data collected during the follow-up, qualitative, and endline data collection in the year 2024. Project funding is guaranteed until December 2024.

## Discussion

### Summary

By implementing a mixed methods approach within a cRCT study design with multiple stakeholders from academia, the private sector, humanitarian organizations, and MoHs and MoEs, we aim to better understand the complex situations in our 4 project countries. We used mixed methods to generate much-needed quantitative data about the WASH infrastructure and behavior in primary health care centers and schools in regions where such data are usually very scarce. At the same time, the qualitative data help to better understand the reasons behind the effectiveness of our intervention and to capture intricacies missed with quantitative data collection. In our opinion, conducting research across disciplines is crucial in humanitarian crisis contexts to guarantee ethical and beneficial outcomes, which policy makers can then use locally.

### Research in Conflict Settings

The demand for research in conflict settings is increasing to support evidence-based interventions and impact evaluations [49]. Unfortunately, this type of research is still rare and the quality of existing studies poor [57]. Apart from obvious challenges such as the researchers’ and participants’ security as well as political instability, additional difficulties might hinder research in these settings [58]. These difficulties include a lack of data, determining the study population, knowing the baseline health status of the study population, displacement of the study population, and issues with logistics [58]. We tried to respond to these difficulties by involving local actors from humanitarian and governmental organizations and the facilities from the project outset. However, the rapidly changing political and security situations in the project countries make it difficult to plan ahead.

### Strengths and Limitations

Our study has some noteworthy strengths: first, with the cRCT, we use the study design with the highest level of evidence [59]. Second, by using mixed methods, we can further strengthen our results by triangulation. Third, the design of the MCHHI is data driven and hence well adapted to promoting sustainability in the contexts in which it was implemented. Fourth, one of the core strengths of this study lies within our inter- and transdisciplinary teams and consortium. By including the humanitarian sector, local authorities, health care personnel, teachers, and students early in the process through regular meetings, the theory of change workshops, and FGDs, we expect to address real needs with our research. Throughout the process of developing the study protocol, we kept consulting with our partners through regular remote meetings and several in-person workshops in the respective countries. This close exchange throughout the project enabled us to rigorously and continuously evaluate the situation in the unstable settings we worked in. Fifth, by conducting this project in 4 different countries, we can gain valuable insights into how the h4h MCHHI needs to be adapted to different contexts and how it can be made more readily available for future interventions in other regions. Finally, we address big data gaps with this study. In Burkina Faso and Mali, the current MoHs actively want to strengthen hygiene, water, and sanitation in health care. However, they lack data about hand hygiene and WASH infrastructure in primary health care to inform their campaigns. This study can inform them directly about current practices and how they can or cannot be influenced with our proposed MCHHI. In schools, the respective MoEs have expressed a keen interest in this study. The findings, which clarify student hand hygiene, are anticipated to be of considerable value. These insights will not only inform about the intervention’s impact but will also guide the MoEs in scaling the intervention across a broader spectrum. By identifying the strengths and weaknesses of the intervention, they can further enhance and replicate the successful aspects while avoiding any identified shortcomings.

Despite the strengths, our study has some limitations. First, we could not choose facilities completely at random. They needed a high probability of remaining accessible to the local partners. Therefore, our results are not fully generalizable and might miss some of the people considered most vulnerable and inaccessible. Second, due to the nature of this study, we could not blind the participants, our implementation partners, or the people conducting the statistical analysis. Therefore, a nonblinding bias cannot be excluded. Third, to maintain a balance between achieving a sample size of institutions for a high enough statistical power and having a sufficient budget to implement hardware interventions in all these institutions, 2 Gravit’eau stations per institution were implemented. In health care centers, we expect an average of 3 health care workers using 1 station and, if accessible to patients, approximately 20 patients per day. In schools, the stations were designed to accommodate up to 100 individuals per hour. Judging from the station’s strategic positioning, we estimate that 250 to 300 students will be using 1 station during a school day. Fourth, we expect a high social desirability bias in the self-reported handwashing and hygiene behavior data. We anticipate having a more realistic and objective view of handwashing and hygiene behavior by complementing the survey data with observation and hand-rinse data. Fifth, the structured observations might be subject to a Hawthorne effect [60]. We aim to counteract this effect by conducting the observations covertly before the official beginning of the study. In health care centers, we chose implementation partners and MoH staff who regularly visit the centers for other supervision activities unrelated to handwashing. This might still change the participants’ behavior, but we expect they did not focus too much on handwashing. We had to inform the head of health care centers before the observations for ethical reasons. Therefore, the possibility that the participants knew why they were observed remains.

### Conclusions

To our knowledge, this is one of the most innovative studies to investigate the effectiveness of an MCHHI on the health determinants of beneficiaries in primary health care centers and schools. With our sound inter- and transdisciplinary methodological approach, we expect to generate results and conclusions that can sustainably impact local policy makers and the humanitarian sector working in the project countries and beyond.

## Appendix

Multimedia Appendix 1 The hands4health consortium.

Multimedia Appendix 2 The hands4health multicomponent hand hygiene intervention.

Multimedia Appendix 3 Description of the variables of the risks, attitudes, norms, abilities, and self-regulation and well-being survey.

Multimedia Appendix 4 Details of hygiene-related absenteeism and health conditions.

## Abbreviations

CFU: colony-forming unit
CONSORT: Consolidated Standards of Reporting Trials
cRCT: cluster randomized controlled trial
FACET: Facility Evaluation Tool for Water, Sanitation, and Hygiene in Institutions
FGD: focus group discussion
h4h: hands4health
KII: key informant interview
MCHHI: multicomponent hand hygiene intervention
MoE: ministry of education
MoH: ministry of health
RANAS: risks, attitudes, norms, abilities, and self-regulation
WASH: water, sanitation, and hygiene
WHO: World Health Organization
