# Supplementary material for: Assessing the Effectiveness of a Multicomponent Intervention on Hand Hygiene and Well-Being in Primary Health Care Centers and Schools Lacking Functional Water Supply in Protracted Conflict Settings: Protocol for a Cluster Randomized Controlled Trial
Source: JMIR Res Protoc. 2024 Apr 3;13:e52959. doi: 10.2196/52959 (PMC11024751; doi:10.2196/52959)
Supplement: Multimedia Appendix 1 [file resprot_v13i1e52959_app1.pdf]

Appendix

1. The hands4health consortium

Table 1: H4h consortium members presented alphabetically with their project responsibilities

Partner name	Main function	Project responsibilities
**Academia**		
Ecole Polytechnique Fédérale de Lausanne (EPFL)<br><br>https://www.epfl.ch/labs/ltqe/	Research partner	- Assessment of disinfectant stability in handwashing water<br>- Passive release disinfection technology<br>- Integration of disinfection in handwashing systems
Palestine Polytechnic University (PPU)<br><br>https://www.ppu.edu/p/	Research partner	- Technology evaluation and choice for local context<br>- Water quality analysis<br>- User-centered design evaluation<br>- Data collection, analysis and quality control
Swiss Tropical and Public Health Institute<br>(Swiss TPH)<br><br>https://www.swisstph.ch/en/	Research partner	- Public health and impact evaluation expertise<br>- Methodology development and implementation of impact evaluation<br>- Technical inputs for the overall project approach<br>- Capacity development<br>- Stakeholder engagement
University of Applied Sciences and Arts Northwestern Switzerland (FHNW)	Research and project coordination partner	- Overall project management and coordination<br>- Engineering and design expertise (Gravit'eau)<br>- Methods development for performance and user acceptance evaluation<br>- Scale up strategy

https://www.fhnw.ch/en/about-fhnw/schools/lifesciences		- Capacity development<br>- Data analysis<br>- Stakeholder engagement<br>- Dissemination strategy
University of Maiduguri (UNIMAID)<br><br>https://www.unimaid.edu.ng/	Research partner	- Local regulatory framework analysis<br>- Stakeholder engagement
**Humanitarian organizations**		
Cesvi<br><br>https://www.cesvi.eu/	Implementation partner	- Lead organization in Palestine<br>- Local market assessment<br>- Management of local supply chain and logistics<br>- Coordination and evaluation of innovations<br>- Data collection<br>- User-centered design<br>- Behavior change implementation and evaluation<br>- Scale up and diffusion strategies<br>- Capacity development and stakeholder engagement
Skat Foundation<br><br>https://skat-foundation.ch/	Project coordination partner	- Knowledge management through documenting and sharing information<br>- Facilitation of learning process and training<br>- Development of online platform<br>- Co-development of scale-up and diffusion strategies
Terre des hommes (Tdh)<br><br>https://www.tdh.org/en	Implementation partner	- Lead organization in Burkina Faso, Mali and Nigeria<br>- Coordination and evaluation of innovations<br>- Local market assessment<br>- Management of local supply chain and logistics<br>- Data collection<br>- User-centered design<br>- Behavior change implementation and evaluation

		- Scale up and diffusion strategies<br>- Capacity development and stakeholder engagement
**Private sector**		
Martin Systems GmbH<br><br>https://www.martin-membrane.de/en/	Hardware supply partner	- Ultrafiltration module manufacturer<br>- Optimization of modules<br>- Quality control and production<br>- Development of interface and business strategy
Ranas<br><br>https://ranas.ch/	Behavior change specialists	- Leading partner for behavior change<br>- Assessment of motivators and barriers for uptake of hand hygiene innovations<br>- Development of behavior change campaign design tool-kit<br>- Training and certification of RANAS experts
