# Supplementary material for: Assessing the Effectiveness of a Multicomponent Intervention on Hand Hygiene and Well-Being in Primary Health Care Centers and Schools Lacking Functional Water Supply in Protracted Conflict Settings: Protocol for a Cluster Randomized Controlled Trial
Source: JMIR Res Protoc. 2024 Apr 3;13:e52959. doi: 10.2196/52959 (PMC11024751; doi:10.2196/52959)
Supplement: Multimedia Appendix 2 [file resprot_v13i1e52959_app2.pdf]

2. The hands4health multicomponent hand hygiene intervention

The multicomponent intervention varies by study country, as it is adapted to the countries' needs, local acceptability of intervention components and tools available for health care centers or schools (Table 1). These intervention components covering hardware, management and monitoring, and behavior change will be applied in at least one of our study countries: (1) a handwashing water recycling station called Gravit'eau, (2) a RANAS behavior change intervention [27], (3) capacity development for regular chlorination and monitoring of drinking water, (4) the Water and Sanitation for Health Facility Improvement Tool (WASH FIT), (5) the preventive maintenance and management of WASH infrastructure at facilities, including water storage, water distribution, handwashing and sanitation and (6) rehabilitation of WASH infrastructure in schools. Following a systemic approach, the interventions were chosen based on the needs and the context identified in workshops applying the theory of change methodology.

Table 1: h4h intervention components applied in each study setting.

	**Primary health care facilities**		**Primary schools**	
Intervention components	**Burkina Faso**	**Mali**	**Nigeria**	**Palestine**
(i) Gravit'eau handwashing station	✓	✓	✓	
(ii) RANAS behavior change	✓	✓	✓	✓
(iii) Chlorination capacity development	✓	✓		✓
(iv) Water and sanitation for health facility improvement tool	✓	✓		
(v) WASH infrastructure management	✓	✓	✓	✓
(vi) WASH infrastructure rehabilitation				✓

One part of the h4h MCHHI is the construction and installation of the Gravit'eau handwashing stations. Workshops were set up and capacity developed in Mali, Burkina Faso and Nigeria to build the Gravit'eau locally. Gravit'eau is a handwashing station which filters and recycles water with gravity [28]. The station does not need to be connected to any electricity source as a manual foot pump operates it. The design of the station is adaptable to the context and can hold up to 90 liters of water. The grey water is collected and treated using gravity with a sand and grease trap and gravity-driven membrane filtration [28, 29]. The membrane module contains an ultrafiltration membrane that removes microorganisms (Cube Mini, Martin Systems) [29]. The

pathogen-free water is stored in a tank until its next use (http://www.graviteau.ch). To keep an acceptable water quality also in terms of color and odor, the water needs to be replaced, depending on the use, about once a month. The filter lifespan is estimated at five to eight years [28].

RANAS is the **r**isks, **a**ttitudes, **n**orms, **a**bilities, and **s**elf-regulation model, developed by Mosler [27]. This model combined well-known social-cognitive theories from health and environmental psychology [30-32] to produce a guide for designing and evaluating behavior change interventions [27]. In brief, it is an easy-to-use method to measure behavioral factors, assess their impact on behavior, design data-driven behavior change strategies, and measure their effectiveness (Figure 1). Several studies demonstrated the usefulness of the RANAS model for investigating handwashing behavior in LMICs [33-37]

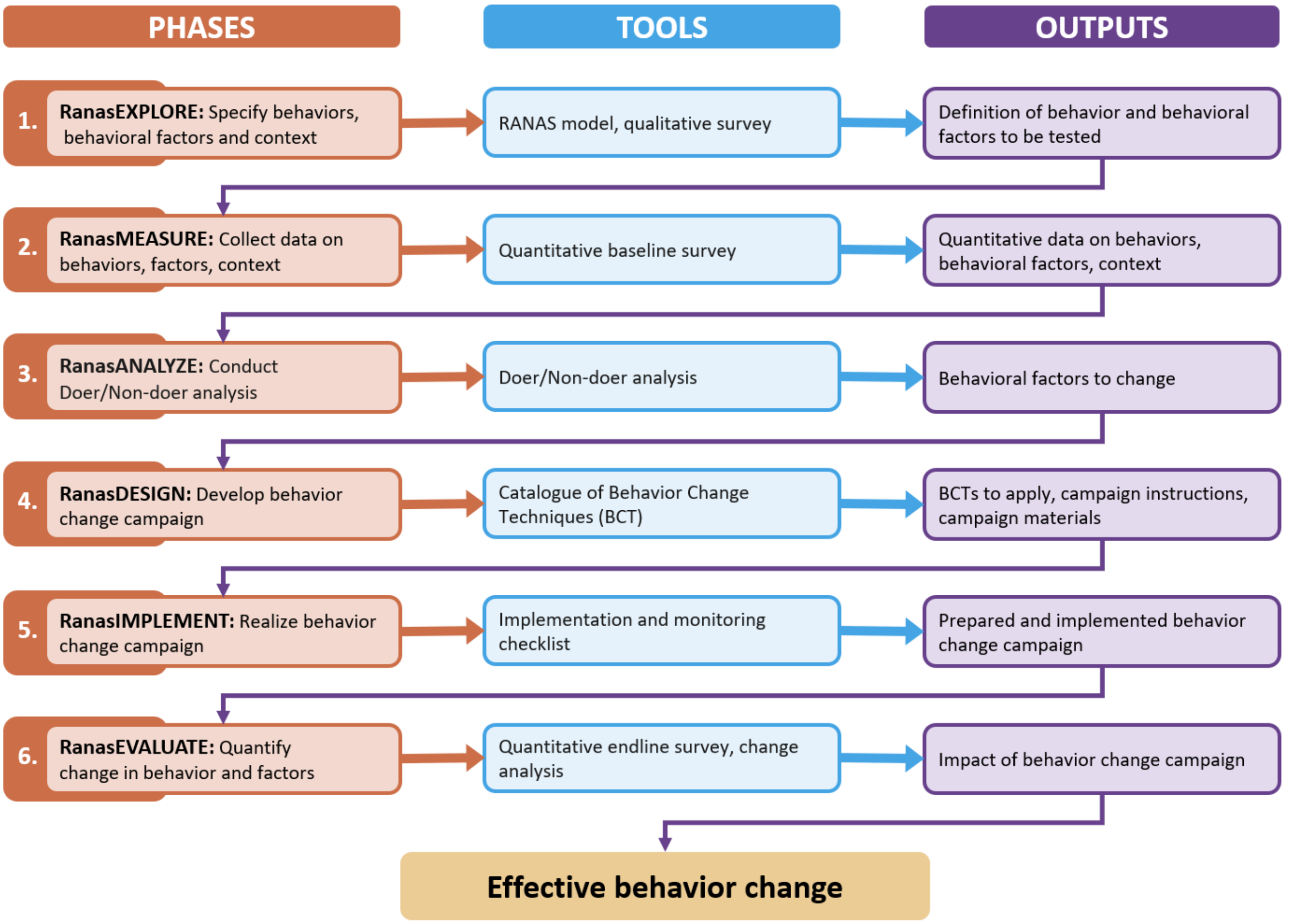

© Copyright Ranas Ltd. Use only under Creative Commons License CC BY-NC-ND 4.0

Figure 1: RANAS behavior change process categorized into the different phases, tools needed and expected outputs.
[1]Doer/Non-Doer analysis: analysis to identify the behavioral factor with the biggest difference between people who report washing their hands and people who do not.
[2]Catalogue of BCT: prior to the data analysis local RANAS experts develop and pilot a catalog of different BCTs for each RANAS factor. Once the factor differing most between doers and non-doers has been identified, the corresponding BCTs can be taken from the BCT catalog.

Chlorination monitoring and management strategies were developed and implemented according to national chlorination standards with the local implementation partners and stakeholders. These strategies were based on a WASH infrastructure questionnaire, which included microbial analysis of the drinking water quality and a detailed assessment of WASH infrastructure. This approach supported the capacity development of the local implementation partners and representatives of health and education authorities. The local authorities will continue with the support of the implementation partners to train the staff of the facilities and review the management systems to ensure that long-term supply chains are functional and that quality control, monitoring and management are guaranteed.

The WASH FIT is a risk-based approach developed by the WHO and UNICEF to improve and sustain WASH and biomedical waste management infrastructure and services in health care facilities in LMICs [26]. This tool should be implemented consistently and regularly, with the objective of first, helping health care center staff and administrators to prioritize, improve and sustain their services and second, supporting national, regional or district-level efforts in health care improvement [26]. The WASH FIT tool includes management and behavioral change interventions for infection prevention and control such as hand hygiene, cleaning of equipment and surfaces, medical waste management and m maintenance and repair of infrastructure [26]. The project includes preventive maintenance, chlorination and Gravit'eau operation, maintenance, and repair in WASH FIT implementation. Support for managing the WASH infrastructure in each institution is carried out according to improvement plans resulting from the WASH FIT process led by the WASH FIT committees from the primary health care facilities. In schools, WASH committees implement similar management approaches. For the preventive maintenance, regular visits of a technician trained and equipped with proper tools under the supervision of the WASH FIT or the School WASH committee is arranged.

In Palestine, various rehabilitation works took place in the schools according to each school's need, based on the infrastructure assessment conducted with the district MoE offices. These activities included the construction of drinking stations, rehabilitation of toilets and handwashing stations, installation of water-saving taps and installation of soap dispensers at drinking and handwashing stations.
