# Supplementary material for: Assessing the Effectiveness of a Multicomponent Intervention on Hand Hygiene and Well-Being in Primary Health Care Centers and Schools Lacking Functional Water Supply in Protracted Conflict Settings: Protocol for a Cluster Randomized Controlled Trial
Source: JMIR Res Protoc. 2024 Apr 3;13:e52959. doi: 10.2196/52959 (PMC11024751; doi:10.2196/52959)
Supplement: Multimedia Appendix 3 [file resprot_v13i1e52959_app3.pdf]

3. Description of the variables of the risks, attitudes, norms, abilities, and self-regulation and well-being survey

Table 1: Variables collected with the RANAS questionnaire survey in primary health care facilities in Burkina Faso and Mali.

Survey section	Variables collected
Socio-demographic information	Sex, highest level of education, age (18-24, 25-49, 50+), marital status, number of people living in the household, profession of spouse
Health care experience	Profession, years of work experience, function in health care center (HCC), unit in HCC, time since last handwashing training
Knowledge	True/false questions about bacteria on clean looking hands, hand hygiene after touching a patient, hand hygiene after touching a patient's object, ability of soap to eliminate bacteria and viruses, hand sanitizer as an alternative to soap, handwashing with soap compared to handwashing with plain water, handwashing being a part of care, main transmission route of pathogens for patients in HCCs, the five moments for hand hygiene (FMFHH)
Handwashing behavior during the FMOHH	Frequency of handwashing with soap, frequency of handwashing with hand sanitizer, frequency of systematic handwashing with soap, frequency of the intention for handwashing with soap, preference for handwashing with soap or hand sanitizer
Risks	Risk of being sick if not respecting the FMFHH, risk of infecting a patient if not respecting the FMFHH, severity of disease if getting sick through a lack of hygiene, severity of disease if a patient is infected because of a lack of hygiene
Attitudes	Ability of FMFHH to prevent infections, effort to collect water for hand hygiene, effort of always washing the hands with soap during the FMFHH, degree of feeling clean if the FMFHH are respected, degree of feeling comfort if FMFHH are respected, degree of feeling protected when respecting the FMFHH, degree of satisfaction with the handwashing stations available in the HCC
Norms	Proportion of team members washing their hands with soap during the FMFHH, appreciation of superiors if staff washes their hands during the FMFHH, feeling of personal obligation to wash the hands during the FMFHH
Abilities	Demonstration of handwashing technique, degree of feeling able to wash the hands during the FMFHH, confidence in always washing the hands during FMFHH (even with problems),

Self-regulation	Awareness of FMFHH during work, knowledge about the location of handwashing stations, knowledge about where to find soap, degree of self-control to wash hands during the FMFHH, frequency of remembering to hand wash during the FMFHH, frequency of time constraints, frequency of water being too slow, frequency of soap not being available, frequency of water not being available, frequency of malfunctioning handwashing stations, having a plan where to wash hands in case of missing water or soap, frequency of forgetting to wash the hands during the FMFHH, degree of feeling engaged to always wash hands during the FMFHH
Gravit'eau handwashing station	Intention to use the Gravit'eau during work and reasons of intention, intention to use the Gravit'eau in private and reasons of intention, confidence in the use of recycled water, degree of feeling good using recycled water, degree of feeling bad using recycled water, confidence in continuation of handwashing if the Gravit'eau is broken
Toilets	Reasons that toilets in HCC cannot be used, frequency of handwashing after urination, frequency of handwashing after defecation
Water in HCC	Availability of drinking water point, availability of water point for handwashing, satisfaction with drinking water, are the basic water needs covered for: drinking, personal hygiene, medical activities, cleaning of the HCC, laundry of the HCC, cooking at the HCC, frequency of water cuts, water quality
Quality of care	What is missing to offer a good quality of care, confidence in personal quality of care offered

Table 2: Variables collected with the combined RANAS and well-being survey in schools in Nigeria and Palestine

**Survey section**	**Variables collected**
Socio-demographic information	Sex, age (7,8,9,10,11,12, and 13), class (third, fourth, fifth, sixth and seventh), number of siblings
Knowledge	A series of true or false questions, including: whether germs can stick to hands; the effectiveness of removing germs by washing hands with water alone; the assumption that soap is not necessary for children when washing their hands; the belief that only adults are at risk from cholera (in Nigeria) and coronavirus (in Palestine); the potential for disease transmission through touching contaminated objects; and the capacity of germs to cause cholera (in Nigeria) and coronavirus (in Palestine)
Handwashing behavior	Frequency of soap availability at school, frequency of handwashing with soap at different situations while at school, frequency of handwashing with soap while at school at two key situations: before eating and after using the toilet
Risks	Risk associated with contracting Cholera or the Coronavirus in the absence of handwashing with soap, particularly in two key situations: severity of these diseases; and the severity associated with the situation when one of the family members contracted Cholera or Coronavirus and the potential impact of the diseases on their lives.
Attitudes	The extent of time, soap, and water consumption during handwashing with soap, the level of hand hygiene achieved after washing with soap, the degree of preference for handwashing in the two key situations, the extent to which handwashing before eating disrupts playtime, the level of perceived disturbance to study time when taking a break to wash hands after using the toilet, the degree of enjoyment of the soap's smell, the degree of discomfort with wet hands, the degree of preference for warm water, and the extend of pleasure when combining play with handwashing using soap.
Norms	Proportion of classmates who wash their hands at the two key situations, the extent to which participants believe their friends and teachers want to see them washing hands with soap, and the degree of guilt felt when neglecting handwashing with soap at the two key situations.
Abilities	Inviting the participant to demonstrate or describe in detail the steps they would take for proper handwashing with soap, and the extent of the participants' belief in their ability to consistently wash hands with soap.

Self-regulation	Participant’s knowledge of when to wash their hands at school, knowledge and awareness of handwashing locations at their school, knowledge of soap locations at their school, self-monitoring of participant’s handwashing habits with soap at the two key situations, and the frequency of skipping handwashing due to the following situations: lack of soap, being busy playing with friends, the handwashing sink is too dirty, or the queue at handwashing station is too long. The frequency of forgetting handwashing with soap at the two key situations.
Satisfaction with the level of access to handwashing services at school	The perceived sufficiency and cleanliness of the water supply for handwashing in school, the perceived quality of the school's handwashing water based on its color and smell, and the perceived clarity and purity of the school's handwashing water, free from suspended solids or dirt. The level of participant’s satisfaction with the handwashing stations provided in their school.
Well-being: physical health	Participant’s perceived frequency of being ill, having headache or tummy-ache, feeling tired or worn-out, and feeling strong and full of energy
Well-being: general feeling	Participant’s perceived frequency of having fun, feeling bored, feeling alone, and feeling scared.
Well-being: self-feeling	Participant’s perceived frequency of feeling proud of themselves, feeling on top of the world, feeling pleased with themselves, and having lots of good ideas.
Well-being: family	Participants’ perceived frequency of feeling fine at home, quarrelling at home, and being stopped by their parents from doing certain things.
Well-being: friends	The frequency of playing with friends, feeling liked by other kids, getting along well with friends, and feeling different from other kids.
Well-being: school	Participant’s perceived frequency of their ease with schoolwork, enjoyment of lessons, concerns about their future, and worries over obtaining poor grades.
