# Supplementary material for: Assessing the Effectiveness of a Multicomponent Intervention on Hand Hygiene and Well-Being in Primary Health Care Centers and Schools Lacking Functional Water Supply in Protracted Conflict Settings: Protocol for a Cluster Randomized Controlled Trial
Source: JMIR Res Protoc. 2024 Apr 3;13:e52959. doi: 10.2196/52959 (PMC11024751; doi:10.2196/52959)
Supplement: Multimedia Appendix 4 [file resprot_v13i1e52959_app4.pdf]

4. Details of hygiene-related absenteeism and health conditions

Table 1: Hygiene-related absenteeism and health condition variables recorded in 24 primary health care facilities, respectively 26 schools per country for Module 4.

**Health care workers and students**	Hygiene-related absenteeism (ie. Diarrhea, cold, influenza, COVID-19, respiratory problems, eye infections, skin infections, HIV/AIDS, hepatitis B or C, other infections)	Record of the event, Number of days absent, Reason for absence, The availability of a medical report for a student's absence instance
**Patients**	Maternal mortality	Record of the event, If known, reason for passing
**Patients**	Postpartum endometritis	Record of the event, Number of days treated, Outcome (healthy, unknown, death)
**Patients**	Stillbirths	Record of the event, If known, reason for passing
**Patients**	Neonatal mortality	Record of the event, If known, reason for passing
**Patients**	Neonatal sepsis[59]	Record of the event, Number of days treated, Outcome (healthy, unknown, death)
**Patients**	Umbilical cord infection	Record of the event, Number of days treated, Type of infection, Outcome (healthy, unknown, death)
**Patients**	Wound infection after stitching of surgical procedures	Record of the event, Number of days treated, Outcome
